# How are systematic reviews used in the planning and design of health technology assessment funded trials?

**DOI:** 10.1186/1745-6215-16-S2-O3

**Published:** 2015-11-16

**Authors:** Sheetal Bhurke, Andrew Cook, Anna Tallant, Amanda Young, Elaine Williams, James Raftery

**Affiliations:** 1 National Institute for Health Research (NIHR), Evaluation, Trials and Studies Coordinating Centre (NETSCC), University of Southampton, Southampton, UK; 2 Wessex Institute, University of Southampton, Southampton, UK; 3 University of Southampton and University Hospital Southampton, Southampton, UK

## Background

Limited evidence exists on how systematic reviews are used in the design of new trials. A study by Jones (2013) showed that 11 out of 48 applications made no reference to a systematic review. Of the 37 trials referencing a systematic review 20 reported their use in the design of the trial.

## Objectives

To replicate and verify Jones' study and explore the reasons why some trials do not refer or use a systematic review. The study also included an updated cohort of NIHR HTA trials to identify any improvements over time.

## Methods

Two cohorts of NIHR HTA randomised controlled trials were included. Cohort I included the same trials as Jones (2006-2008). Cohort II included NIHR HTA trials funded in 2013. Data extraction was undertaken independently by two reviewers and results were presented using descriptive statistics.

## Results

Justifying the need for new primary research using a systematic review is not always feasible. Our study found nine (19%) and three (9%) trials from cohort I and II respectively where a systematic review was not referenced. Although our findings were similar to Jones, we found all nine trials had a justifiable reason for not referring to a systematic review.

## Conclusions

The results of this study demonstrate how 85% of NIHR HTA trials use systematic reviews to inform the design and planning of a new trial. Systematic reviews play an important role in the development of clinical trials and the implications of this will be discussed.

